# *In vivo* AGO-APP for cell-type- and compartment-specific miRNA profiling in the mouse brain

**DOI:** 10.1016/j.crmeth.2025.101267

**Published:** 2025-12-29

**Authors:** Surbhi Kapoor, Andrea Erni, Francesca Vincenzi, Beatrice Tessier, Vasika Venugopal, Gunter Meister, Alexandre Favereaux, Harold Cremer, Christophe Beclin

**Affiliations:** 1 Aix-Marseille Université, Centre National pour la Recherche Scientifique (CNRS), Institut de Biologie du Développement de Marseille (IBDM), Marseille, France; 2 University of Bordeaux, CNRS Interdisciplinary Institute for Neuroscience, UMR 5297, Bordeaux, France; 3 Regensburg, Center for Biochemistry (RCB), University of Regensburg, Regensburg, Germany

**Keywords:** miRNA, Argonaute, cortical glutamatergic neurons, olfactory bulb interneurons, Dicer, TNRC6, T6B, postsynapse

## Abstract

AGO-APP through the expression of the T6B peptide permits the isolation of Ago-bound microRNAs (miRNAs). Here, we present the generation and characterization of two transgenic mouse lines that enable AGO-APP to be performed *in vivo*. First, we generated mice for CRE-dependent T6B expression throughout the cell. Using this line, we performed AGO affinity purification (AGO-APP) in olfactory bulb (OB) inhibitory interneurons and cerebral cortex excitatory neurons. Bioinformatic analysis validated the high reproducibility of the approach. It also demonstrated that, despite global miRNome conservation between the two cell types, a set of miRNAs, including the miR-200 family and the miR-183/96/182 cluster, is massively enriched in OB interneurons, which aligns with previous observations. In the second mouse line, T6B is fused to the postsynaptic protein PSD95. Isolation of T6B-PSD95 fractions from OB and cortical neurons identified specific sets of postsynapse-enriched miRNAs. Gene ontology analyses confirmed that these miRNAs preferentially target mRNAs related to synaptic functions.

## Highlights

• We present two T6B-expressing transgenic mouse lines for *in vivo* AGO-APP studies
• CRE-dependent T6B expression enables cell-type-specific Ago-miRNA isolation
• OB interneurons and cortical excitatory neurons exhibit distinct miRNA signatures
• PSD95-T6B fusion uncovers postsynaptic miRNA enrichment related to synaptic genes

## Motivation

Deciphering miRNA expression with cell-type and subcellular resolution remains a major challenge, as current analyses rely predominantly on *in vitro* approaches that fail to recapitulate physiological conditions. In this study, we present two transgenic mouse lines that enable the identification and quantification of Argonaute-bound miRNAs in defined cell types of the mouse brain. This approach further allows the characterization of miRNAs within specific subcellular compartments.

## Graphical abstract

**Figure:**
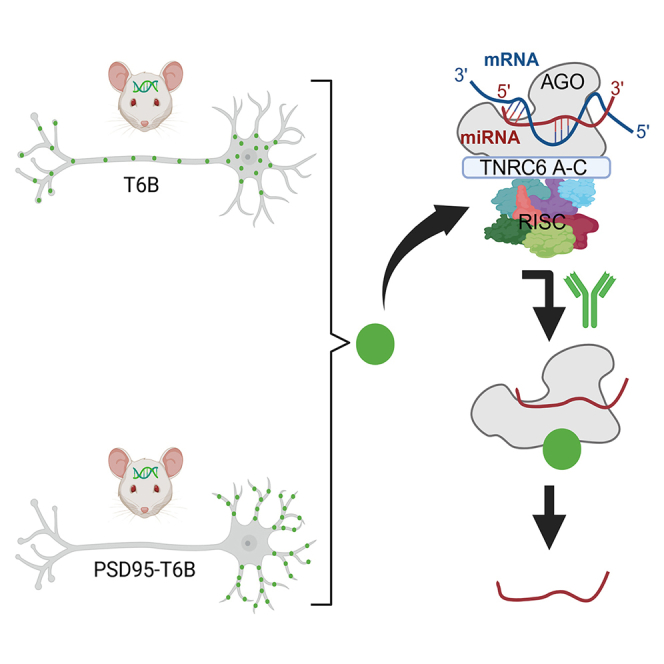

## Teaser

Kapoor et al. present two transgenic mouse lines enabling *in vivo* AGO affinity purification (AGO-APP) through expression of the T6B peptide. These models allow cell-type-specific and postsynapse-associated miRNA profiling, revealing distinct miRNA populations and synaptic enrichment patterns across olfactory bulb interneurons and cortical excitatory neurons.

## Introduction

MicroRNAs (miRNAs) are small non-coding RNA molecules that regulate gene expression by binding to complementary sequences on target mRNAs. Although the exact number of mammalian miRNAs remains debated, recent estimates suggest about 2,300 in humans^1^ and 2,200 in mice.^2^ Interference with the function of the miRNA pathway, for example through genetic inactivation of the miRNA-generating ribonuclease Dicer, causes severe developmental and tissue homeostasis defects, underscoring its essential physiological role.^3^

miRNA/mRNA interactions are highly promiscuous. A single miRNA can bind to many mRNAs, and a specific mRNA can present binding sites for several miRNAs. Over 60% of transcripts are predicted miRNA targets.^4^ Therefore, detailed information about the expression of miRNAs and mRNAs in a given cell type is essential to infer functional interactions. While mRNA expression can now be profiled at tissue and single-cell levels using RNA sequencing and spatial transcriptomics, miRNA expression analysis remains challenging, particularly in complex tissues or rare cell types *in vivo*.

Current miRNA expression analyses require relatively large input amounts, and single-cell sequencing remains highly experimental.^5^^,^^6^ Imaging methods, such as *in situ* hybridization, are limited by miRNAs small size and promiscuity.^7^ Fluorescence-activated cell sorting (FACS) of dissociated primary cells before sequencing has been used but depends on the availability of suitable markers.^8^ Moreover, morphologically complex cell types, like for example mature neurons, are refractory to intact dissociation. Finally, not all expressed miRNAs are bound to Argonaute (AGO) and incorporated into the RNA-induced silencing complex (RISC), indicating that only a subset is functionally active.^9^^,^^10^^,^^11^

Beyond their cytoplasmic functions, there is evidence that miRNAs have specialized roles when targeted to specific cellular compartments like axons^12^^,^^13^ or the nucleus.^14^^,^^15^^,^^16^ However, the specific presence and function of miRNAs is best documented for the postsynapse, for which several studies suggest a role in regulating local translation, and consequently synaptic plasticity.^17^^,^^18^^,^^19^

Given this high degree of expression and functional complexity new technologies are needed to overcome the existing roadblocks. T6B is an 80-amino-acid (aa) peptide derived from the human TNRC6B protein, which links the AGO-miRNA-mRNA complex with the downstream mRNA degradation machinery in RISC.^20^ Although non-functional, T6B retains the ability to bind all AGO proteins involved in the miRNA pathway and can be used to capture AGO-bound miRNAs in pull-down experiments in a process called AGO-APP.^21^ We recently showed that transgenic expression of T6B in *Drosophila melanogaster* enables precise isolation and analysis of miRNAs from neural stem cells, neurons, and glia, leading to the discovery of a module of miRNAs that cooperatively preserve neural progenitors from premature differentiation.^22^

We present here two transgenic mouse lines allowing the CRE-dependent expression of T6B, either throughout the cell or specifically targeted to the postsynapse. We use these lines for the direct and reliable isolation and analysis of miRNAs from specific neuron types in the vertebrate brain and provide proof of principle that the subcellular localization of miRNAs at the postsynapse can be addressed by this approach.

## Results

### Sustained expression of T6B in neuronal progenitors does not perturb postnatal neurogenesis

First, we investigated how lack of miRNAs impacts neurogenesis. Using a conditional Dicer mutant mouse line (Dicer-floxed^23^), it was demonstrated that Dicer function is required for the development of specific cell types, in particular the cortical glutamatergic neurons in the mouse embryo.^24^ We investigated whether dicer function is also important for postnatal neurogenesis.

**Figure 1:**
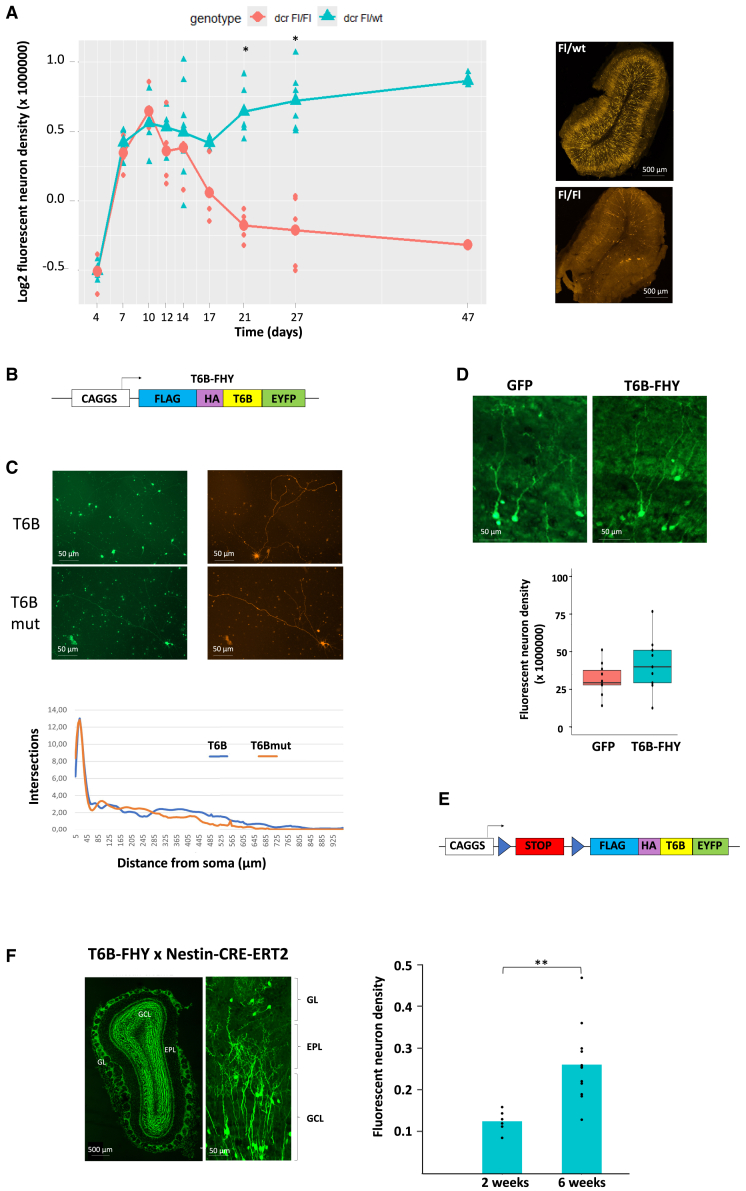
Sustained expression of T6B in neuronal progenitors does not perturb postnatal neurogenesis (A) Left: time course (log_2_ scale) of recombined neuron density in OB sections after CRE electroporation in the SVZ of P1 mice heterozygous (green) or homozygous (red) for a conditional mutant allele of dicer. Each symbol represents one animal; 3–4 sections were quantified per individual. ∗*p* ≤ 0.05. Right: representative mosaic images of OB coronal section from dicer heterozygous (Fl/wt) or homozygous (Fl/Fl) animals. (B) Scheme of the pCX-T6B-FHY plasmid. (C) Top: green and red channel images of cortical neurons 7 days after nucleofection with pCX-T6B-FHY (top) or pCX-T6Bmut-FHY (bottom) plus Ai9 plasmid.^34^ Bottom: Sholl analysis (*n* = 11 cells per group) showed no significant difference in neurite growth between plasmids (linear mixed-effects model). (D) Representative anti-GFP-stained OB sections 5 weeks after P1 electroporation with pCX–GFP (left) or pCX–T6B–FHY (right). Boxplot: density of fluorescent neurons per OB section (× 10^6^) after electroporation with pCX-GFP (red) or pCX-T6B-FHY (green) plasmid. Cell density in individual animals is shown as a black dot (3–4 sections quantified per individual). (E) Scheme of the construct used to generate the conditional T6B-FHY-expressing transgenic mouse line. *LoxP* sites, encompassing a STOP cassette, are indicated as triangles. (F) Left: OB section stained with anti-GFP antibody 5 weeks after tamoxifen injection in a P1 T6B-FHY animal (left). The high magnification image (right) shows individual T6B-FHY-expressing neurons in the granule cell layer (GCL) and the glomerular layer (GL). EPL, external plexiform layer. Right: density of fluorescent neurons in OB sections of T6B–FHY × Nestin-CRE-ERT2 animals at 2 and 5 weeks after tamoxifen injection. Six (2 weeks) and 12 (5 weeks) animals were treated (three OB sections quantified per animal). ∗∗*p* value ≤0.01.

Neural progenitors (NPs) located in the subventricular zone of the forebrain ventricles (SVZ) generate permanently a high diversity of inhibitory interneurons for the olfactory bulb (OB). These new neurons form functional synapses around 5 weeks after their birth.^25^ This system can be easily manipulated by postnatal *in vivo* electroporation of the ventricular wall.^26^^,^^27^ Importantly, the neurogenic system was shown to implicate the function of specific miRNAs, acting at different steps of the process, from neuron production and phenotypic determination to synaptic function. For example, miR-124 favors the exit from a neural progenitor state,^28^ whereas miR-9 controls subsequent proliferation.^29^ MiR-7^30^ and miR-124^31^ regulate fate determination, while miR-200^32^ and miR-132^33^ participate in final maturation of new neurons. We investigated the level of dependency of this neurogenic system on a functional miRNA pathway using the conditional dicer mutant mouse line.^23^ During the first 2 weeks after CRE electroporation in the lateral SVZ, comparable numbers of recombined neurons were detected in the OB granule cell layer of control (CRE:+/fl) and dicer-deficient (CRE:fl/fl) animals (Figure 1A). Over the following weeks, the number of recombined neurons almost doubled in control mice due to ongoing neuron production by recombined NPs in the SVZ. In contrast, there was a significant drop in the density of recombined neurons in dicer−/− animals, until the almost total loss of all recombined cells at 7 weeks post-electroporation (Figure 1A). We conclude that a functional miRNA pathway is essential for the generation of new neurons in the SVZ-OB system.

Previous *in vitro* work provided evidence that binding of T6B to AGO can disrupt the RISC complex and interfere with the function of the miRNA pathway.^21^ *In vivo*, de-repression of several target mRNAs and defects in homeostasis of skeletal muscles and heart have been observed. However, changes in miRNA levels were not detected in these studies.^35^^,^^36^ More recently, AAV-mediated transduction of a T6B vector in Purkinje cells of mice was shown to inhibit miRNA function and alter the proper development of these neurons, leading to their death.^37^

In light of these findings, we decided to investigate whether in our conditions T6B expression impacts neurogenesis in the mouse forebrain. First, we checked whether T6B expression is detrimental to primary cortical neuron development *in vitro*. We compared the growth of neurites in cortical neurons electroporated with a vector expressing T6B or a mutated, non-functional T6B peptide (T6Bmut).^21^ Both peptides were fused to FLAG and HA tags, as well as the fluorescent protein EYFP, and cloned downstream of a pCAGGS promoter^38^ (Figure 1B), giving rise to pCX-T6B-FHY and pCX-T6Bmut-FHY plasmids. Seven days after electroporation, Sholl analysis showed that dendritic arborization between neurons expressing T6B or T6Bmut was indistinguishable (Figure 1C).

We then investigated if the expression of T6B may interfere with neurogenesis *in vivo*. SVZ NPs were electroporated at P1 with the pCX-T6B-FHY. Five weeks after electroporation EYFP-positive neurons in the OB did not reveal obvious morphological alterations or significant differences in the number of granule cells compared to control neurons electroporated with a pCX-GFP plasmid^38^ (Figure 1D), indicating that any potential T6B-induced perturbation was too minor to affect the proper generation and integration of new cells.

Based on these observations, we generated a transgenic mouse line allowing the CRE-dependent expression of T6B-FHY (Figure 1E) under the CAGGS promoter. The construct was integrated into the Rosa26 locus^39^ by homologous recombination. Resulting transgenic mice were bred to a Nestin-CRE-ERT2 transgenic line^40^ to drive temporal controlled recombination in postnatal SVZ NPs upon tamoxifen injection at P1. Two weeks after induction, the granule cell layer and the glomerular layer (GL) of the OB were strongly colonized by EYFP-positive cells with the typical morphology of young granule and periglomerular neurons (Figure 1F). At 5 weeks post-electroporation, the density of newborn neurons more than doubled (Figure 1F), comparable to the increase observed in Dicer +/fl mice and strikingly different from the cell loss observed in Dicer mutants (Figure 1A). Thus, other than after total loss of the miRNA pathway induced by Dicer KO (Figure 1A), neurogenesis in the SVZ-OB system proceeds at normal levels in the presence of T6B-FHY expression, indicating that any potential interference with miRNA activity is minor, not obviously impacting neuronal integration and survival.

### miRNA isolation from brain tissue by *in vivo* AGO-APP

**Figure 2:**
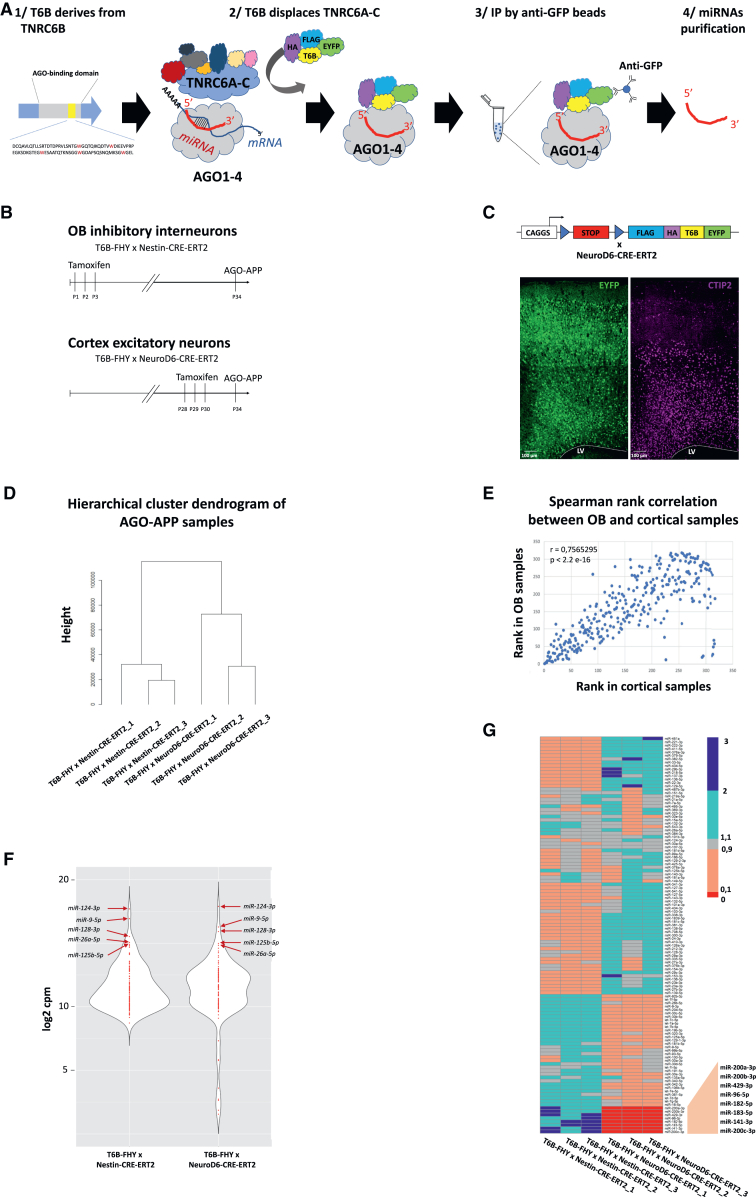
miRNA isolation from brain tissue by *in vivo* AGO-APP (A) Scheme of the T6B peptide and AGO-APP procedure. (B) Timeline for generating AGO-APP samples from OB inhibitory interneurons and cortical excitatory neurons. (C) Mosaic image showing EYFP expression in cortical glutamatergic neurons across motor cortex layers (left) after tamoxifen injection in T6B–FHY × NeuroD6-CRE-ERT2 P34 mice; CTIP2 marks layers V and VI neurons (right). (D) Hierarchical clustering of AGO-APP miRNA-seq samples from T6B-FHY × Nestin-CRE-ERT2 and T6B-FHY × NeuroD6-CRE-ERT2 shows tight cell-type clustering. (E) Spearman correlation of mean cpm reads between OB interneurons (Y) and cortical neurons (X) for miRNAs with sum of cpm reads of all six samples >100; r = 0.7565, adjusted *p* value <2.2E-16. (F) Violin plots of log_2_ mean-normalized miRNA expression (cpm) for miRNAs with sum of cpm reads from the six samples >5,000. The top five expressed miRNAs per condition are labeled. (G) Heatmap of normalized values (computed by dividing the normalized cpm read counts by the mean of all samples) for all miRNAs with a sum of cpm reads of all six samples >5,000; close-up highlights the eight miRNAs strongly overexpressed in OB versus cortical neurons. See also Table S1.

Binding of T6B to AGO disrupts RISC by displacing TNRC6 proteins from the complex (Figure 2A). Our previous work in *Drosophila melanogaster* demonstrated that the miRNA:AGO:T6B-FHY complex can be efficiently pulled down with anti-GFP nanobodies.^22^ Subsequently, miRNA is released and can be analyzed by sequencing. This approach was used to generate a high-resolution map of miRNA expression in drosophila neural stem cells, neurons, and glial cells.^22^

We aimed at using this innovative approach to provide insight into the miRNA expression in different types of vertebrate neurons. Indeed, due to their complex morphology and their tight contact with other cells, isolation of intact neurons from vertebrate brain tissue and analysis of their cytoplasmic content remain highly challenging.^41^ Consequently, information about neuronal-subtype-specific expression of miRNAs is currently limited. AGO-APP is suited to overcome this problem as T6B-FHY can be expressed under the control of suited promoters in specific cell types for directly isolating miRNAs from tissue homogenates, without the need for cell isolation. We used this approach to compare miRNA expression between GABAergic inhibitory neurons and glutamatergic excitatory neurons.

To isolate miRNAs from GABAergic neurons, we induced CRE recombination in SVZ progenitors by the Nestin-CRE-ERT2 driver, subsequently leading to T6B-FHY expression in inhibitory OB interneurons (Figures 1E and 1F). Three tamoxifen injections were performed at P1, P2, and P3, and 5 weeks later, when newborn neurons derived from these NPs arrived in the OB and were fully mature, OBs were dissected, homogenized, and miRNAs were isolated by AGO-APP (Figure 2B).

To drive expression of T6B-FHY in mature glutamatergic projection neurons, we used a well-characterized inducible NeuroD6-CRE-ERT2 line, which drives CRE recombination into cortical excitatory neurons, including layer V and VI neurons expressing CTIP2 (Figure 2C^42^). For AGO-APP, tamoxifen was injected three times at P28–P30, and cortical tissue containing mature T6B-FHY-expressing neurons was harvested 4 days later (Figure 2B).

Subsequently, miRNAs pulled down by the AGO-APP procedure were sequenced using a modified version of the AQ-seq protocol,^43^ optimized for samples with low RNA concentration. Hierarchical clustering of samples based on miRNA sequencing reads showed that the three OB replicates and the three samples issued from cortex clustered tightly together respectively (Figure 2D), demonstrating high reproducibility of the approach.

After normalization to “counts per million” (cpm) and the exclusion of miRNAs poorly expressed over all samples (sum of cpm reads from all samples less than 100; Table S1), we compared the global miRNA expression pattern between the two cell types using Spearman rank correlation (Figure 2E). This analysis resulted in a correlation coefficient (r) of 0.7565295, indicating an overall conservation of the miRNomes. This overlap is well exemplified by the observation that the five miRNAs showing strongest expression in GABAergic neurons were also the highest present in glutamatergic neurons (miR-124-3p, miR-9-5p, miR-128-3p, miR-125b-5p, and miR-26a-5p) (Figure 2F).

However, despite obvious overlap, the cluster analysis suggested differences in miRNA content from both neuron types (Figure 2D). For a graphical comparison of both miRNomes, we built a heatmap focusing on the 117 most strongly expressed miRNAs (sum of cpm reads from all samples greater than 5,000). Heatmap representation of the homogenized data allowed to pinpoint a set of eight miRNAs, belonging either to the miR-200 family (miR-200a-3p, miR-200b-3p, miR-429-3p, miR-141-5p, and miR-200c-3p) or to the miR-183/96/182 cluster (Figure 2G), that showed striking differential expression, with strong presence in OB neurons but quasi absence from cortical neurons. This observation was fully consistent with our previous findings in raw OB tissue,^32^ further validating the AGO-APP approach. Moreover, these eight miRNAs have been functionally implicated in the control of neurogenic events. Indeed, the miR-200 family is involved in the maturation of OB interneurons and the development of olfactory sensory neurons,^32^^,^^44^ while the miR-183/96/182 cluster controls maturation and maintenance of retinal photoreceptors.^45^^,^^46^

We then performed a DESeq2 statistical analysis to systematically determine differentially expressed miRNAs between the two types of neurons. As expected, strongest differential expression was observed for the abovementioned set of eight miRNAs overexpressed in the OB (Table 1). Moreover, we identified a total of 209 miRNAs for which the *p*-adjusted value was lower than 1%. Implementing a fold change threshold of 2 and a base mean of 500, this number was reduced to 62, among which 30 were found overexpressed in cortical neurons (Table 1). Importantly, this list of cortex-enriched miRNAs comprised miR873a-5p, miR218-5p, miR137-3p, miR27-3p, miR-185-5p, and miR-433-3p, six miRNAs for which previous GWAS analyses showed an association with neurological disorders involving the cortex, such as autism spectrum disorder,^47^ schizophrenia,^48^^,^^49^^,^^50^ bipolar disorder,^51^^,^^52^ or depressive symptoms.^53^

**Table 1:** Results of a DESeq2 analysis comparing the expression of miRNAs in OB interneurons and excitatory cortical neurons

	baseMean	log2FoldChange	lfcSE	*p* value	*p* adj
**Overexpressed in OB interneurons**					

miR-183-5p	4,66183713	10.2631557	0.36637166	2.60E-173	1.75E-170
miR-96-5p	1,563.70102	9.85545685	0.40370272	1.37E-131	1.84E-129
miR-182-5p	5,169.69564	9.53412553	0.34454259	5.39E-170	1.81E-167
miR-200c-3p	1,108.09195	9.07264993	0.3803197	1.71E-126	1.91E-124
miR-141-3p	1,896.07816	9.06928571	0.33240498	2.17E-164	4.85E-162
miR-200b-3p	5,836.19201	7.4786064	0.28465722	3.65E-153	6.11E-151
miR-429-3p	5,441.6094	7.41531176	0.32970703	3.83E-113	3.67E-111
miR-200a-3p	11,310.9171	6.92777945	0.31481078	1.84E-108	1.55E-106
miR-92b-3p	1,057.46525	3.42757066	0.21751717	6.46E-57	4.82E-55
miR-20a-5p	601.169773	3.10698059	0.27262826	5.17E-31	2.89E-29
miR-17-5p	619.506539	2.39787941	0.24444812	1.66E-23	6.97E-22
miR-15b-5p	508.545204	2.18657729	0.24381821	5.58E-20	1.78E-18
let-7d-3p	538.441738	1.96478868	0.31378391	8.22E-11	9.59E-10
miR-484	719.327873	1.95974651	0.24277917	1.56E-16	4.04E-15
miR-204-5p	2,806.75239	1.84510456	0.20077702	1.00E-20	3.36E-19
miR-25-3p	737.091075	1.71290929	0.2475498	1.24E-12	2.20E-11
miR-9-3p	24,198.9774	1.61734783	0.22463072	1.81E-13	3.68E-12
let-7a-5p	10,221.6995	1.5142682	0.23924748	8.29E-11	9.59E-10
let-7f-5p	15,785.3655	1.4427159	0.30039163	5.54E-07	3.75E-06
miR-19b-3p	1,348.962	1.39327394	0.20250046	2.27E-12	3.62E-11
let-7c-5p	23,389.5382	1.38332519	0.19788718	1.08E-12	1.96E-11
miR-485-3p	612.248643	1.38214147	0.21546796	5.58E-11	6.69E-10
miR-30c-5p	23,089.6242	1.35174472	0.24276875	1.14E-08	1.05E-07
miR-26b-5p	1,998.38526	1.3029657	0.33208983	3.50E-05	0.00017134
miR-30b-5p	9,481.15735	1.24903865	0.21313096	2.07E-09	1.99E-08
let-7b-5p	11,053.9647	1.23069911	0.20261038	5.75E-10	5.93E-09
miR-320-3p	1,021.09035	1.14819994	0.31797984	0.00014481	0.00064349
miR-125a-5p	8,818.9733	1.07403416	0.23311791	2.19E-06	1.35E-05
miR-129-1-3p	1,661.51409	1.07086947	0.21736954	4.49E-07	3.14E-06
miR-350-3p	721.927616	1.05326893	0.28977651	0.00014725	0.00064576
let-7d-5p	5,103.84616	1.00826499	0.22474924	4.14E-06	2.46E-05
miR-384-5p	896.950211	1.00433066	0.31465173	0.00078106	0.00292787

**Overexpressed in cortical neurons**					

miR-873a-5p	717.436755	−3.513534	0.28512463	5.95E-36	3.99E-34
miR-451a	4,804.18394	−2.91146144	0.2596646	3.84E-30	1.98E-28
miR-136-5p	5,051.1175	−2.38226214	0.26867724	1.12E-19	3.40E-18
miR-129-5p	3,922.112	−2.18871742	0.31789006	1.02E-12	1.90E-11
miR-218-5p	5,751.94551	−2.00995929	0.26307943	4.44E-15	1.06E-13
miR-383-5p	588.024934	−1.9923327	0.31586303	5.57E-11	6.69E-10
miR-22-3p	8,695.03733	−1.9790903	0.26428779	1.40E-14	3.23E-13
miR-137-3p	4,498.98785	−1.92847282	0.2847317	2.88E-12	4.38E-11
miR-221-3p	4,878.82707	−1.85297178	0.19219498	1.28E-22	4.78E-21
miR-377-3p	912.625118	−1.73987879	0.25882912	4.65E-12	6.63E-11
miR-379-5p	1,178.30338	−1.71993116	0.27448104	9.09E-11	1.03E-09
miR-185-5p	789.300947	−1.71389086	0.27763465	1.79E-10	2.01E-09
miR-382-5p	2,509.67415	−1.69898798	0.29257991	1.56E-09	1.52E-08
miR-744-5p	525.326008	−1.66053874	0.27264495	3.12E-10	3.37E-09
miR-29b-3p	14,294.1527	−1.64155244	0.25785069	5.44E-11	6.69E-10
miR-376a-3p	14,428.7306	−1.62688289	0.23807521	3.06E-12	4.57E-11
miR-154-5p	800.78465	−1.50479922	0.24530812	2.69E-10	2.95E-09
miR-411-5p	1,963.89352	−1.45489517	0.20964497	1.37E-12	2.36E-11
miR-434-5p	1,628.04975	−1.42892281	0.20887046	2.79E-12	4.36E-11
miR-222-3p	3,133.62121	−1.42264328	0.20051986	4.70E-13	9.21E-12
miR-326-3p	502.627486	−1.4036276	0.25516039	1.33E-08	1.19E-07
miR-330-5p	611.753635	−1.35425069	0.26936039	1.88E-07	1.43E-06
miR-33-5p	3,095.22815	−1.3073424	0.26164055	2.33E-07	1.72E-06
miR-139-5p	4,067.93144	−1.29512154	0.19721624	2.10E-11	2.82E-10
miR-29c-3p	6,477.46508	−1.21390249	0.23694326	1.27E-07	1.01E-06
miR-153-3p	3,618.32954	−1.15990599	0.32943278	0.00017897	0.0007698
miR-127-5p	1,186.54231	−1.12577119	0.27313957	1.77E-05	9.18E-05
miR-433-3p	623.083681	−1.11937252	0.20415291	2.05E-08	1.79E-07
miR-541-5p	2,693.48469	−1.06089516	0.28952386	0.00012554	0.00056159
miR-27b-3p	5,357.8878	−1.05903029	0.177264	1.22E-09	1.21E-08

Only results for miRNAs exhibiting an adjusted *p* value lower than 1%, a fold change greater than 2, and an expression baseMean greater than 500 are shown.

Overall, these data show that *in vivo* AGO-APP using the transgenic mouse line is highly reproducible and suited for determining miRNA expression in specific neuron subtypes at high resolution.

### Analyzing miRNA expression at the postsynapse

The above-described experiments demonstrate that the expression of T6B in transgenic mice allows the capture of Argonaute-bound miRNAs from different neuron types. However, besides their well-described role in general mRNA translational control, there is strong evidence that miRNAs have specialized roles when targeted to specific cell compartments, like for example the postsynaptic aspect of glutamatergic synapses.^17^^,^^18^^,^^19^

**Figure 3:**
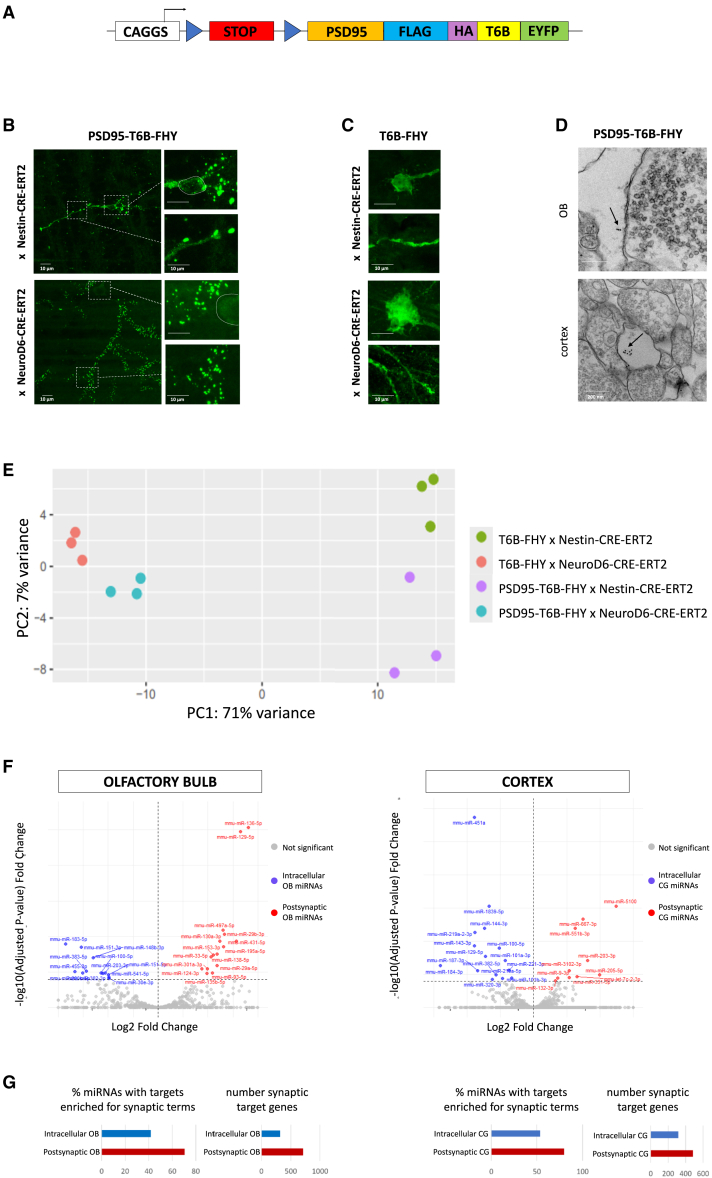
*In vivo* AGO-APP to analyze miRNA expression at the postsynapse (A) Scheme of the PSD95-T6B-FHY transgene. (B) Confocal images (P34 animals) of PSD95-T6B-FHY-expressing OB interneurons (top) and cortical glutamatergic neurons (bottom) stained with anti-GFP. Close-ups show minimal cytoplasmic signal in cell bodies (top) and punctate peripheral staining in dendrites (bottom). (C) In P34 animals, OB interneurons and cortical neurons expressing T6B-FHY without PSD95 show homogeneous staining, lacking punctate synaptic localization in the cell body (top) or within dendrite (bottom). (D) Electron microscopy (EM) images after anti-GFP gold bead labeling of OB interneurons (top) or cortical projection neurons (bottom) synapses expressing PSD95-T6B-FHY (P34 animals). Arrows show groups of beads in the postsynaptic compartment. (E) Principal-component analysis (PCA) of miRNA-seq data from AGO-APP samples issued from the four transgenic lines (three replicates each): T6B-FHY × Nestin-CRE-ERT2, T6B-FHY × NeuroD6-CRE-ERT2, PSD95-T6B-FHY × Nestin-CRE-ERT2, and PSD95-T6B-FHY × NeuroD6-CRE-ERT2. (F) Volcano plot for DESeq2 results showing miRNAs differentially present at the postsynapse in OB interneurons and cortical projection neurons. miRNA enriched at the postsynapse or throughout the cell are colored in red and blue, respectively. (G) GO analysis of predicted mRNA targets of postsynaptically enriched miRNAs in OB interneurons (left) and cortical neurons (right). Left: percentage of miRNAs with predicted mRNA targets significantly enriched in synaptic genes; right: cumulative number of predicted synaptic mRNA targets.

In mammals, postsynaptic density protein 95 (PSD95) is confined to the postsynaptic compartment of excitatory synapses^54^ where it critically regulates synaptic function and dendritic spine morphology.^55^ PSD95 has been used to efficiently transport fused protein to the postsynapse, without altering synaptic morphology or physiology.^56^ Based on these properties, we generated a second transgenic line, in which T6B-FHY was fused to PSD95 (Figure 3A). First, we studied subcellular localization, in either OB inhibitory neurons with the Nestin-CRE-ERT2 driver or cortical excitatory neurons, using NeuroD6-CRE-ERT2 (Figure 3B). Analyses of EYFP fluorescence at P34 in both lines validated that PSD95-T6B-FHY showed always the striking punctate staining typical of synaptic localization, whereas labeling in cell bodies (surrounded by a dotted line) was very faint, different from the diffuse but strong labeling throughout the cell observed for T6B-FHY alone (Figure 3C, but see Figure 1F). PSD95-driven localization at postsynapse was validated using electron microscopy of immunogold stained material from OB and cortex, which showed clusters of electron-dense particles mainly confined to postsynaptic structures (Figure 3D).

We then verified that the ectopic expression of the PSD95-T6B transgene does not impair the ability of neurons to undergo homeostatic synaptic scaling, a type of plasticity in which PSD95 supports synapse strengthening and spine growth.^57^

To elicit homeostatic synaptic scaling, we applied 2-μM TTX to organotypic hippocampal slices derived from PSD95-T6B-FHY × NeuroD6-CRE-ERT2 animals at DIV14 for 48 h and then performed whole-cell patch-clamp recordings on pyramidal neurons to assess the impact on synaptic AMPAR-mediated spontaneous synaptic currents relative to untreated slices. In agreement with previous reports,^58^^,^^59^^,^^60^ TTX treatment induced a significant shift in the distribution of sEPSC amplitudes and inter-event intervals (Figures S1A–S1C), suggesting both an increase in synaptic AMPARs and an increase in the number of active synapses.

To assess the impact of TTX treatment on spine size, we infused through the patch pipette biocytin in the recorded neurons. Subsequent analysis of dendritic spine morphology showed that TTX treatment induced an enlargement of the spine head area within the normal range (0.48 μm^2^ versus 0.34 μm^2^, *p* value<0.0001) (Figures S1D and S1E),^60^ further confirming that hippocampal pyramidal neurons expressing PSD95-T6B-FHY maintain ability to undergo homeostatic plasticity at the expected level.

Following these experimental validations, we performed AGO-APP and small RNA sequencing on either OB or cortical tissue dissected from the PSD95-T6B-FHY mouse lines and compared the resulting data with those previously obtained with the T6B-FHY lines. Principal-component analyses (PCAs) demonstrated strong clustering of all biological replicates (three per condition) underlining again the high reproducibility of the approach (Figure 3E). Neuron type explained most of the observed variability, whereas variability explained by cell compartment (intracellular versus postsynapse) was considerably lower. For both neuron types, analyses of differential expression between T6B-FHY and PSD95-T6B-FHY isolates identified small sets of miRNAs showing significant different expression between both compartments (Figure 3F), indicating preferential postsynaptic enrichment of a subset of miRNAs. Interestingly, 6 among 15 miRNAs enriched in the OB postsynaptic fraction (miR-138,^61^ miR-153,^62^ miR-135,^63^ miR-124,^60^ miR-29a/b,^64^ and miR-129-5p)^65^ and 2 among the 10 cortical postsynaptic high miRNAs (miR-9^61^ and miR-132^66^) have been previously associated to synaptic function in other studies. Finally, we predicted potential target mRNAs of the miRNAs enriched in the OB and cortical PSD95-T6B-FHY fractions, using the TargetScan algorithm.^67^ Gene ontology analyses of this dataset revealed that the AGO-APP postsynaptic fractions of OB and cortex neurons were enriched for miRNAs targeting mRNAs that are linked to synaptic terms (Figure 3G).

Altogether, these data provide strong evidence that PSD95-T6B-FHY is a potent tool for the isolation of postsynaptic miRNAs.

## Discussion

miRNAs emerged as important regulators of gene expression in the cell. Collectively they are required for most, if not all, cellular processes, as demonstrated by the severe phenotypic alterations following entire pathway inactivation.^3^ Our finding that Dicer deficiency prevents the generation of new interneurons in the postnatal OB further underscores their essential role.

However, beyond the general conclusion that the miRNA pathway is required for a process to occur, the precise mechanism by which miRNAs regulate is still largely unclear, mainly due to conceptual and technical limitations. Indeed, experiments addressing the role of specific miRNAs generally induce only minor phenotypes,^68^^,^^69^^,^^70^ not compatible with an all-or-nothing function of individual miRNA-target interactions. In agreement, several recent studies provided evidence that entire sets of miRNAs cooperate to control the expression of a particular gene or a regulatory network.^22^^,^^70^

Technically, analyzing the expression and function of miRNA at the cellular level is highly challenging. For example, the amount of needed input material is currently not compatible with routine single-cell sequencing, and histological approaches are still challenging. Moreover, cell isolation *in vivo* depends generally on tissue microdissection and dissociation followed by cell sorting. Disruption of complex cells like neurons during dissociation will lead to miRNA loss. Finally, functional studies by inhibiting the expression of specific miRNAs are often complicated by the fact that many miRNA loci are located within protein coding genes or produced from more than one genomic location.^71^

The situation is even more complicated. Indeed, it has been reported that not all miRNA expressed in a given cell are involved in regulation but that “silent” and “active” miRNAs coexist at a given time point.^9^^,^^10^^,^^11^ Thus, to launch subsequently complex functional studies, simply isolating and analyzing the entire complement of cellular miRNAs might not provide sufficiently conclusive information and may lead to the prediction of multiple non-functional interactions. Finally, there are considerable data indicating transport of miRNAs to, and function in, specific subcellular compartments like axons, nucleus, or synapse. Unbiased investigation of miRNAs showing such subcellular localization *in vivo* is currently impossible.

*In vivo* AGO-APP represents a tool to overcome many of these limitations. First, transgenic expression of T6B allows the isolation of miRNAs from complex cellular environments like brain tissue. Due to the expression of T6B as a fluorescent fusion protein, cell types to be analyzed can be studied at high resolution, allowing the correlation of morphological and sequencing data. Also, as the miRNA-AGO-T6B complex forms in the intact cell, disruption of the cytoplasm does not lead to loss of material but is an intrinsic part of the isolation approach. Finally, *in vivo* AGO-APP is a benchtop approach, not requiring sophisticated multistep manipulations, specialized equipment, or expertise in FACS, allowing the recovery of sufficient amounts of miRNAs for reliable sequence analysis, leading to meaningful data.

AGO-APP is based on the immunoprecipitation of T6B together with AGO-bound miRNAs that can be considered to be the “active fraction” in a background of all expressed miRNAs.^10^ Ago-APP presents similarities with technologies based on the immunoprecipitation of miRNAs bound to a tagged AGO2 protein. These AGO2 traps have been expressed either from an exogenous functional transgene^72^ or, in case of HEAP-seq^73^ and SAP-seq,^37^ from the modified endogenous Ago2 locus. These approaches allow the simultaneous pull-down of both the miRNAs and the targeted mRNAs. However, other than our T6B approach, these technologies do modify the amount of functional Ago2 in the cell.^37^ While in case of SAP-seq, this reduction does not apparently affect the miRNA pathway in the cerebellar Purkinje cells^37^; it cannot be excluded that in other cell-types the situation may be different. Moreover, whereas such approaches do only allow to pull-down miRNAs bound to AGO2, AGO-APP, which is based on the controlled expression of a transgenic exogeneous peptide, allows the cell-type-specific isolation of miRNAs bound to all four AGO proteins.^21^

Here, we isolated miRNAs from two spatially separated cell populations, cortical excitatory neurons and OB interneurons. We find overall similarity between the AGO-bound miRNA pools, suggesting global conservation of the regulatory pathway in neurons. However, the high resolution of AGO-APP allowed us to resolve quantitative variations in miRNA numbers between both cell types and also led to the identification of an OB-specific subset of miRNAs that might act in concert to fulfill specific roles in these neurons.

Finally, our approach enables us to systematically address the presence of miRNAs in a cellular sub-compartment. Indeed, there is a vast literature describing postsynaptic miRNAs and their specific activities during homeostasis and plasticity.^19^ Addressing such functions in a physiological context is challenging, if not impossible, with the available technologies. However, as shown here, T6B can be specially targeted to the postsynapse, allowing the isolation and analyses of miRNAs enriched in this compartment. We found that miRNAs enriched at the postsynapse, defined by the intracellular-to-postsynaptic ratio, differed markedly between the two analyzed cell types, despite overall miRNome conservation. Moreover, the identified postsynaptic miRNAs showed limited overlap with those reported previously in the literature, suggesting that postsynaptic miRNA targeting is cell-type-specific and shaped by the physiological environment rather than intrinsic miRNA properties.

AGO-APP represents a technical advance in understanding the expression and mode of action of miRNA. Using two different CRE drivers, this technology allowed us to compare AGO-bound miRNAs expression in two given neuron types. This analysis was made possible because T6B expression does not, or only marginally, affect the development of the analyzed neurons. However, recent work showed that infecting the cerebellar Purkinje cells with T6B-expressing AAV particles leads to developmental defects and death of these neurons.^37^ Moreover, the authors described a detrimental effect of T6B expression on neurite growth in *in vitro* cultured cortical neurons. The fact that in our conditions we did not observe such an effect of T6B on the development of the same cells suggests that the effect of T6B depends on experimental conditions. Nevertheless, these observations demonstrate that, in order to extend the scope of AGO-APP technology to other cell types in the brain and other tissues, it is essential to verify that neither the T6B inducing CRE driver nor the experimental conditions are associated with developmental and/or behavioral defects in the analyzed cells.

We have also shown that AGO-APP enables the study of specific miRNA enrichment at postsynapses in living animals. Therefore, AGO-APP will allow the local binding of synaptic miRNAs to AGO as a function of neuronal activity and plasticity to be studied, for example during learning and memory. Finally, using alternative molecular tags, other than PSD95, AGO-APP could also probe miRNA activity in other cell compartments for which local functions have been suggested, such as the axon or nucleus.

### Limitations of the study

Whereas our control experiments reveal no effect of T6B expression on neurogenesis, we cannot exclude that T6B expression reduces the level of miRNA regulation, altering the expression of a subset of genes.

In this work, we applied AGO-APP to compare AGO-bound miRNA expression between OB GABAergic and cortical glutamatergic neurons and between the intracellular and the postsynaptic compartments. Although these data are consistent with previous findings, we do not provide an in-depth functional analysis. Indeed, such direct experimental validations are difficult to design, especially for validating the enrichment of miRNAs at the postsynapse of specific neurons under *in vivo* conditions. Alternatively, indirect validation may be envisaged by, for example, correlating the postsynaptic enrichment of miRNAs presumably involved in plasticity with artificial change in neuronal activity.

The AGO-APP technology presented in this manuscript enables immunoprecipitation of AGO-bound miRNAs and offers several advantages over concurrent technologies based on immunoprecipitation of AGO proteins, including the possibility to analyze the targeting of miRNAs to specific compartments of the cell. In contrast, we believe that co-isolation of target transcripts with the AGO-bound miRNAs, as has been shown for other approaches, may be problematic. Indeed, it is likely that the displacement of TNRC6 protein by T6B weakens the miRNA-mRNA association in the cell.^74^ However, we did not specifically address this point.

## Resource availability

### Lead contact

Requests for further information, resources, and reagents should be directed to and will be fulfilled by the lead contact, Christophe Beclin (christophe.beclin@univ-amu.fr).

### Materials availability

Plasmids and strains generated in this study are available upon request from the lead contact, Christophe Beclin (christophe.beclin@univ-amu.fr).

### Data and code availability

• The sequencing datasets generated and analyzed during this study are available at the functional genomics data collection, ArrayExpress, accession number E-MTAB-15124 (https://www.ebi.ac.uk/biostudies/arrayexpress/studies/E-MTAB-15124).
• This study does not report original code.
• Any additional information required to reanalyze the data reported in this paper is available from the lead contact upon request.

## Acknowledgments

The Cremer lab has been supported by the ANR (MicroRNAct, ANR-17-CE16-0025; Uncoding, ANR-21-CE16-0034; Miniature, ANR-21-CE13-0003) and the 10.13039/501100002915Fondation pour la Recherche Médicale (Program Equipe FRM). S.K. received a PhD fellowship from Neuroschool Marseille from Aix Marseille University. A.E. received a postdoc fellowship from the 10.13039/501100001711Swiss National Science Foundation (SNSF).

We thank Lena Vilvandre for animal genotyping, Marie-Catherine Tiveron for confocal imaging, Nathalie Core for manuscript review, Mathieu Letellier for his help in designing the physiological experiments, and Sophie Chauvet for assistance with the graphical abstract. We acknowledge the animal facilities and the IBDM imaging facility, part of France-BioImaging (https://ror.org/01y7vt929), which are supported by ANR-24-INSB-0005 FBI BIOGEN. Electron microscopy was performed at the PiCSL-FBI core facility (IBDM, AMU-Marseille), part of France-BioImaging (ANR-10-INBS-04), and the Marseille Imaging Institute, an Excellence Initiative of Aix Marseille University A∗MIDEX, a french “Investissements d’Avenir” programme (AMX 19 IET 002). The funders had no role in study design, data collection and analysis, decision to publish, or preparation of the manuscript. We also thank members of the Meister lab for providing the T6B-FHY construct and valuable advice.

## Author contributions

C.B. and H.C. designed the study and wrote the paper. S.K., A.E., B.T., and V.V. performed the experiments. F.V. achieved several bioinformatic analyses. A.F. supervised the electrophysiological experiments. G.M. helped in designing the *in vivo* AGO-APP protocol.

## Declaration of interests

The authors declare no competing interests.

## STAR★Methods

### Key resources table

**Table:** 

REAGENT or RESOURCE	SOURCE	IDENTIFIER
**Antibodies**		

Rabbit anti-GFP	Thermo Fisher Scientific	PA1-28521; RRID:AB_1956473
Rat Anti-CTIP2 IgG	Abcam	Ab18465; RRID:AB_2064130
Donkey Anti-rabbit Alexa Fluor™ 488	Thermo Fisher Scientific	A21206; RRID: AB 2535792
Donkey anti-Rat 568	Abcam	AB175475; RRID:AB_2636887

**Chemicals, peptides, and recombinant proteins**		

poly-*l*-lysine-hydrobromide	Merck	P6282-5MG
Neurobasal medium	Thermo Fisher Scientific	21103049
B27 Supplement, serum-free	Thermo Fisher Scientific	17504044
AEBSF, READY MADE SOLUTION 100 MM IN H20	Merck	SBR00015-1ML
DTT (DITHIOTHREITOL)	ROTH	6908–1
Ethanol	VWR	20821.296
GLYCINE	EUROMEDEX	26-128-6405-C
Glyco Blue Coprecipitant	Thermo Fisher Scientific	AM9515
Na_2_EDTA	VWR	20302.236
NP-40	Thermo Fisher Scientific	28324
Paraformaldehyde 32%	EUROMEDEX	EM-15714
PBS	EUROBIO	CSOPBS01-08
Pierce™ Bovine Serum Albumin standard 2mg/ml	Thermo Fisher Scientific	23209
Potassium chloride (KCl)	ROTH	3904.2
Sodium Dodecyl Sulfate (SDS) 20%	EUROMEDEX	EU0660-B
SODIUM FLUORIDE (NaF)	Merck	PHR1408-1G
sunflower seed oil	Merck	S5007-250ML
Tamoxifen free base	Merck	T5648-1G
TBE BUFFER, 10X	Thermo Fisher Scientific	15581044
Tris-Hcl 1M pH7	Interchim SA	AYO661
TRIZOL REAGENT	Thermo Fisher Scientific	15596026
Tetrodotoxin citrate (TTX)	Tocris Bioscience	1069/1
streptavidin Alexa 647	Thermo Fisher Scientific	S32357
Biocytin	Tocris Bioscience	3349
ACSF	Tocris Bioscience	3525
D-Gluconate	Merck	S2054
HEPES	Merck	H4034
EGTA	Merck	324626
Proteinase K (PCR grade)	Roche product	3115801001
*E. coli* poly(A) polymerase	New England Biolabs	M0276S
GoTaq® G2 Flexi DNA Polymerase	Promega	M7808
PHUSION HIGH-FIDELITY DNA POLY MERASE, 100U	Thermo Fisher Scientific	F530S
SuperScriptIII First Strand cDNA Super Mix	Thermo Fisher Scientific	18080–400
T4 RNA Ligase 1	New England Biolabs	M0204S

**Critical commercial assays**		

ChromoTek GFP-Trap® Agarose	Proteintech	gta-100
COSTAR(R) SPIN-X(R) CENTRIFUGE TUBE	Merck	CLS8163-100EA
Kit HS DNA BioAnalyzer	Agilent Technologies	5067–4626
MiniSeq High Output Reagent Kit (75-cycles)	Illumina	FC-420-1001
miRCURY LNA RT Kit	Qiagen	339340
miRCURY LNA SYBR Green PCR Kit	Qiagen	339346
miRCURY LNA miRNA PCR Assay	Qiagen	339306
NOVEX 6% TBE GEL 1.0MM 10W	Thermo Fisher Scientific	EC6265BOX
Nucleofector ® II	Lonza	N/A
**Nunc™ Lab-Tek™ II Chamber Slide™**	Thermo Fisher Scientific	154461PK
Fluoromount-G™	Thermo Fisher Scientific	00-4958-02

**Deposited data**		

miRNA sequencing data from Ago-APP samples obtained with the T6B and PSD95-T6B expressing transgenic mouse lines	this paper	ArrayExpress: E-MTAB-15124

**Experimental models: Organisms/strains**		

T6B-FHY and PSD95-T6B-FHY expressing mouse lines	this paper	N/A
Nestin-CRE-ERT2 mouse line	Battiste et al.^40^	N/A
NeuroD6-CRE-ERT2 mouse line	Agarwal et al.^42^	N/A
CD-1® IGS mouse line	Charles River, France	Strain Code: 022
Dicer mutant mouse line	The Jackson Laboratory	B6.Cg-Dicer1tm1Bdh/J
Ai14 mouse line	The Jackson Laboratory	B6.Cg-Gt(ROSA)26Sor/J

**Oligonucleotides**		

randomized 5′ adapter GUUCAGAGUUCUACAGUCCGACGAUCNNNN	Kim et al.^43^	N/A
v-dT20-RA3 RT Primer GCCTTGGCACCCGAGAATTCCA TTTTTTTTTTTTTTTTTTTTV	this paper	N/A

**Recombinant DNA**		

pCX-CRE	Morin et al.^38^	N/A
pCX-GFP	Morin et al.^38^	N/A
pCX-T6B-FHY	this paper	N/A
pCX-T6Bmut-FHY	this paper	N/A
plasmid Ai9	Addgene; Madisen et al.^34^	22799; RRID: Addgene_22799

**Software and algorithms**		

ImageJ	Schneider et al.^75^	https://ImageJ.net/ij/; RRID: SCR_003070
R version 4.4.2	The R Foundation	https://www.r-project.org/; RRID: SCR_001905
Package ggplot2 of R	https://ggplot2-book.org/	https://ggplot2.tidyverse.org; RRID: SCR_014601
R Package lme4	https://doi.org/10.32614/CRAN.package.lme4	https://github.com/lme4/lme4/; RRID: SCR_015654
R Package lmertest	https://doi.org/10.32614/CRAN.package.lmerTest	https://github.com/runehaubo/lmerTestR; RRID:SCR_015656
R Package dplyr	https://doi.org/10.32614/CRAN.package.dplyr	https://dplyr.tidyverse.org; RRID: SCR_016708
R Package pheatmap	https://CRAN.R-project.org/package=pheatmap	https://CRAN.R-project.org/package=pheatmap; RRID: SCR_016418
R Package DESeq2	Love et al.^76^	https://rdocumentation.org/packages/DESeq2/versions/1.12.3; RRID: SCR_016533
R Package RcmdrPlugin.coin	The R Foundation	https://CRAN.R-project.org/package=RcmdrPlugin.coin
R Package GenomicAlignments	Lawrence et al.^77^	https://bioconductor.org/packages/GenomicAlignments; RRID: SCR_024236
R Package clusterProfiler	https://yulab-smu.top/contribution-knowledge-mining/	https://bioconductor.org/packages/clusterProfiler/; RRID: SCR_016884
ZEN Blue	Zeiss	RRID: SCR_013672
TargetScanMouse 8.0	Agarwal et al.^67^	https://www.targetscan.org/mmu_80/; RRID: SCR_010845
Clampex 10.3	MOLECULARDEVICES	https://www.moleculardevices.com/; RRID: SCR_011323
Minianalysis software 6.0	Synaptosoft	https://www.synaptosoft.com/; RRID: SCR_002184

### Experimental model and study participant details

#### Animals

All mouse experiments were approved and performed according the guidelines of French Ethical committee according to the European rules and regulations (Authorization number #25923–2020081214524914 v1). Mice were group-housed in regular cages under standard conditions on a 12 h light-dark cycle. The day of birth was defined as postnatal day 0 (P0). For each experiment involving animals (preparation of primary neurons, *in vivo* electroporation in P1 pups, or tamoxifen injection), each experimental condition involved entire litters. As the mouse lines used in this study produced males and females at an equal sex ratio, both sexes were equivalently represented in each experiment.

### Method details

#### *In vitro* culture and electroporation of mouse cortical neurons

Cortical neurons were prepared from E15 wild-type embryos (CD1) and cultured as previously described.^78^ They were electroporated using *Nucleofector II* device from Lonza (Program setting A-023). Following nucleofection, the cells were plated for seven days in a two-wells Lab-Tek chamber, previously coated with poly-L-lysine. After seven days of culture, the neurons were fixed for 15 min in PFA 4% and rinsed 3 times in PBS. Mosaic images were acquired using an apotome fluorescence microscope (Axioplan2, ApoTome system, Zeiss, Germany). Analysis of neurites growth was performed using the Sholl method (SNT plugin of ImageJ^75^).

#### *In vivo* electroporation and transgenic mice

*in vivo* electroporation experiments were performed on P1 pups.^26^

The construct used to generate the T6B-FHY transgenic mouse line described in this paper was derived from the Ai14 plasmid by replacing the tomato transgene by T6B-FHY.^21^ The resulting construct was subsequently used to derive the plasmid that was introduced into the genome of the PSD95-T6B-FHY mouse line by fusing the PSD95 coding sequence from rat to the 5′ end of T6B-FHY.

Transgenesis was performed at the SEAT transgenesis center (CNRS, Villejuif, France). The 2 constructs were integrated into the genome of 129/SvPas ES cells by homologous recombination at the ROSA-26 locus. Recombined ES cells were identified by PCR screening and subsequently microinjected into blastocytes of pseudogestant C57BL/6N females. The chimeras were then back-crossed to C57BL/6N animals.

#### Brain sections preparation, immunohistochemistry for optical imaging

Animals were deeply anesthetized with an overdose of xylasin/ketamine then intracardially perfused with 4% paraformaldehyde (w/v; PFA) in PBS. The brains were dissected out and further fixed overnight at 4°C in the same fixative. Fifty micrometer wide floating sections were processed as previously described,^27^ blocked in PBS supplemented with 0.3% Triton and 5% fetal bovine serum for 1 h at room temperature and incubated at 4°C overnight in the blocking solution supplemented with primary antibody. After washing, sections were incubated for 2 h at room temperature in the blocking solution supplemented with a fluorophore-conjugated secondary antibody. Sections were mounted onto Superfrost Plus slides with Mowiol. Images were acquired using an apotome fluorescence microscope (Axioplan2, ApoTome system, Zeiss, Germany) or a laser confocal scanning microscope (LSM780, Zeiss, Germany).

#### Cell counts and statistical analysis

The density in fluorescent neurons was counted differently depending on the experiment. For Figure 1A, the number of fluorescent neurons per OB section was counted manually. However, because this time-course experiment included multiple independent electroporation experiments performed on different litters which can cause variations in the efficiency of electroporation, we normalized the average number of fluorescent neurons per OB section with the average number of remaining fluorescent cells in the SVZ region^29^ which estimates the efficiency in electroporation. For Figure 1E, the number of fluorescent neurons per OB section was counted manually. For Figure 1F the number of fluorescent neurons per section was too high to be counted manually, therefore the quantity in fluorescent neurons was estimated automatically using ImageJ by counting the number of fluorescent pixel. In all cases, the density was subsequently deduced by dividing the estimated number of fluorescent neurons in a given section by the section area, expressed in pixel x pixel, counted automatically with ImageJ.

#### Electron microscopy

Mice underwent perfusion with 4% paraformaldehyde (PFA) in phosphate-buffered saline (PBS). Following perfusion, brains were dissected out and 1 mm^2^ pieces of brain tissue were chopped. The pieces of brain were fixed with OsO4 2% in PBS for 1 h at 4°C, then washed in PBS, dehydrated with an ethanol gradient and embedded in Epon resin. Polymerization was then performed for 48 h at 60°C. The blocs were cut using an ultramicrotome (Leica UC7, Leica, Netherlands) to obtain 90 nm thick sections collected on Nickel grids. The grids were first treated for 1–3 min with a saturated solution of sodium metaperiodate, then rinsed for less than 5 min with 1% Triton X-100 in TBS. Next, the grids were incubated for 1 h in 5% BSA with 0.1% fish gelatin skin, followed by an overnight incubation with a chicken anti-GFP primary antibody (diluted 1/250 in TBS for PSD95, 1/125 for T6B). Then the grids were rinsed four times in TBS for 5 min, incubated 1h at 37°C in a secondary antibody (Anti-chicken coupled with 10 nm gold bead) diluted 1/30 in TBS, and washed four times in TBS for 5 min. Finally, the grids were treated with 2.5% Glutaraldehyde in 0.05 M CaCo for 10 min. Images were captured using a Transmission Electron Microscope (Tecnai G2, ThermoFisher, USA) operating at 200 kV. Imaging was facilitated by a Veleta camera (Olympus, Japan).

#### AGO-APP

Olfactory bulb or cortex tissues were dissected from T6B or PSD95-T6B expressing mouse brains and immediately fixed in 1 mL 4% paraformaldehyde (PFA) for 10 min at 25°C. Fixation was quenched in 245 mM glycine for 5 min at 25°C and samples were washed twice with ice-cold phosphate-buffered saline (PBS) for 5 min at 25°C.

Fixed tissue samples were then lysed in 1 mL of lysis buffer containing 150 mM KCl, 25 mM Tris-HCl (pH 7.5), 2 mM EDTA, supplemented freshly with 1 mM NaF, 0.5% NP-40, 1 mM DTT, and 1 mM AEBSF. Cell lysis was performed by sonication using a Vibra Cell sonicator (10 cycles of 1 min and 20 s each; 10 s on, 10 s off; 34% amplitude; 4°C). The lysate was clarified by centrifugation at 15,000 × g for 15 min at 4°C.

Total protein concentration was quantified using the Pierce BCA Protein Assay Kit (Thermo Fisher Scientific, Product Number: 23227), and the lysate was adjusted to a final concentration of 2000 μg in a total volume of 1000 μL.

For each immunoprecipitation (IP), 25 μL of GFP-TRAP Agarose (GTA) beads were used. The beads were blocked with 1% BSA for 2 h at 4°C, followed by two washes with ice-cold PBS, and centrifuged for 2 min at 2500 × g. A total of 950 μL (1900 μg) of cleared lysate was added to the washed beads, and the mixture was incubated for 1 h at 4°C with shaking or on a rotator wheel.

The beads were then washed five times with wash buffer containing 1 M NaCl, 50 mM Tris-HCl (pH 7.5), 5 mM MgCl2, supplemented with freshly added 1 mM NaF (Sigma-Aldrich, Product Number: PHR1408-1G), 0.01% NP-40, 0.1–1 mM DTT (Sigma-Aldrich, Product Number: 3483 12-3), and 0.1–1 mM AEBSF (Sigma-Aldrich, Product Number: SBR00015-1ML). During the final wash step, the beads were transferred to a new tube precoated with PBS for at least 1 h to prevent bead sticking. After the final wash, the beads were washed once more with ice-cold 1X PBS.

#### RNA analysis

IP samples were supplemented with 50 μL of 4% SDS in 0.1 M NaHCO_3_, and incubated at 50°C for 10 min with shaking at 700 rpm. The supernatant was collected, and the bead release was repeated to ensure complete recovery. Proteinase K (PCR grade, Roche, Product Number: 03115801001) was prepared at 20 mg/mL in PK buffer and pre-incubated at 37°C for 20 min to inactivate potential RNases. A total of 100 μL of the prepared Proteinase K solution was added to each sample (input and IP), and the mixture was incubated overnight at 65°C with shaking at 1000 rpm. RNA was then extracted with 1 mL of TRIzol reagent following the manufacturer protocol. At the end of the process the RNA pellets were resuspended in 12 μL of nuclease-free water.

To estimate the miRNA concentration in the AGO-APP samples a qRT-PCR was systematically performed according to the manufacturer’s instructions (Qiagen). Briefly, 3 μL of the RNA samples were reverse transcribed. The resulting complementary DNA (cDNA) was diluted 1:10, and 3 μL of the diluted cDNA was used per well for subsequent qPCR analysis using a let-7a-5p targeting probe.

#### Library preparation and miRNAs sequencing

The remaining 9 μL of the AGO-APP samples were first polyadenylated according to the manufacturer (NEB) instructions. Following enzyme deactivation (65°C for 20 min), RNA precipitation with ethanol and resuspension in 2.5 μL of water, a 5′ randomized adapter^43^ was ligated to the polyadenylated miRNAs using T4 RNA-Ligase 1 according to manufacturer (NEB) instructions in a final volume of 10 μL. For this ligation step the adapter concentration was adapted to the amount of miRNA in the sample as estimated by the qRT-PCR previously realized. Adapting the adapter concentration allows to favor the amplification of the miRNA containing molecules. Bellow, the rule we followed:

CT of let-7a-5p 5′ adapter concentration.

14-17 0.18 μM.

18-19 0.05 μM.

19-20 0.01 μM.

21-25 0.003 μM.

Subsequently 3μL of the ligation reaction was directly reverse-transcribed using the v-dT20-RA3 oligonucleotide to prime the reaction. Then the library was amplified by PCR performed on 5 μL of the ligation reaction using the Illumina primers RP1 and an RPIx index primer and following the program: 98° for 1 min - X times (98°C for 10 s, 58°C for 30 s, 72°C for 15 s) - 72°C for 10 min. The number X of cycles was previously determined through pilot PCR reactions performed on 1 μL of the reverse transcription reaction under the same amplification conditions but applying a variable number of cycles: 18, 21 and 25. The products of the PCR reaction were loaded on a 6% polyacrylamide TBE gel, from which the desired band of approximately 165 bp was extracted and purified. After purification, the concentration of all samples of the library was analyzed on an Agilent 2100 Bioanalyzer, according to the manufacturer’s instruction. Equal amounts of each sample were mixed for sequencing on an Illumina Miniseq instrument, according to the manufacturer’s instruction.

#### Organotypic slice culture, tamoxifen and TTX treatment

Organotypic hippocampal slice cultures were prepared from PSD95-T6B expressing mouse brains. Animals at postnatal days P6 were quickly decapitated and brains placed in ice-cold Gey’s balanced salt solution under sterile conditions. Hippocampi were dissected out and coronal slices (350 μm) were cut and cultured as previously described.^60^ The medium was replaced every 2–3 days until slices were used at DIV14 for electrophysiology experiment. At DIV10, medium was complemented with 54μM tamoxifen. At DIV14, slices were incubated or not with 2μM TTX (Tetrodotoxin citrate) for 48h before electrophysiology experiment.

#### Morphological analysis in organotypic slices

Organotypic slices of PSD95-T6B mice used for electrophysiology experiment and patched with biocytin were fixed overnight in PBS containing 4% paraformaldehyde and 4% sucrose, quenched for 15 min with 50 mM NH4Cl in PBS, then blocked and permeabilized for 2h with 1% biotin-free BSA, 1% Triton X-100 in PBS. Slices were incubated overnight in 0.28 nM streptavidin Alexa 647, mounted between coverslip and glass slide in fluoromount and then observed by confocal microscopy. Biocytin positive neurons were observed under a confocal microscope (Leica DM6, CFS TCS SP8, Germany), using a 63X/1.40 N.A. oil immersion objective, and fluorophore excitation with the laser line 647 nm. Image stacks of 1024 x 1024 pixels (corresponding to areas of 72.7 × 72.7 μm, pixel size 71 nm) with a z-step of 0.3 μm were acquired across several microns through the slice (scanning frequency 400 Hz, pinhole at 1 Airy disk). Maximum intensity projections were created under ImageJ. Area spine head analysis is performed with the plug-in SpineJ^79^ under ImageJ.

#### Electrophysiological recordings

Whole-cell patch-clamp recordings were carried out at room temperature in pyramidal neurons from organotypic hippocampal cultures, placed on the stage of a Nikon Eclipse FN1 upright microscope equipped with a motorized stage and two manipulators (Scientifica). Pyramidal neurons expressing PSD95-T6B were imaged with DIC and identified by visualizing the GFP fluorescence. The recording chamber was continuously perfused with ACSF bubbled with 95% O2/5% CO2 containing (in mM): 125 NaCl, 2.5 KCl, 26 NaHCO3, 1.25 NaH2PO4, 2 CaCl2, 1 MgCl2, and 25 glucose. The internal solution contained (in mM) 130 K-D-Gluconate, 7 KCl, 10 HEPES, 0.05 EGTA, 2 MgATP, 2 Na2-ATP, 0.5 Na-GTP, and 13 Biocytin. AMPAR-mediated spontaneous currents (sEPSCs) were recorded at −70 mV for >5 min, digitized at 20 kHz amplifier and filtered at 2 kHz using the Multiclamp 700B (Axon Instruments) and acquired using the Clampex software. The series resistance Rs was left uncompensated, and recordings with Rs higher than 30 MΩ were discarded. Analysis of sEPSCs recordings was made using minianalysis software. The micropipettes were made from borosilicate glass capillaries, with a resistance in the range of 4–6 MΩ.

### Quantification and statistical analysis

#### Statistics

Statistical analyses between two experimental conditions in Figures 1A, 1E, 1F and 4E were performed with the non-parametric exact Wilcoxon test using the RcmdrPlugin.coin package in R.

Results of cortical neurons neurite growth obtained using the Sholl method in Figure 1C were analyzed by a linear mixed effects model approach using the lme4 and lmerTest packages in R.

In Figures S1B and S1C, differences between conditions were analyzed by the Kolmogorov-Smirnov test.

#### Bioinformatic analysis

The fastq files generated by the sequencing process were analyzed, first using the Galaxy website to trim the reads (Trim-Galore method) and to align the reads to the mm10 version of the mouse genome for generating the bam files (BWA method), and then using the GenomicAlignments package of R^77^ to count the miRNA reads. Differential expression was analyzed using the DESeq2 package of R.^76^ To assess the enrichment in GO terms among a list of predicted target genes we used the enrichGO function of the clusterProfiler package of R. Dendrogram and heatmap were generated in R using the stats and pheatmap packages, respectively. Graphs were generated in R using the ggplot2.

## Supplemental information

Document S1. Figure S1

Table S1. miRNA cpm counts of all 12 Ago-APP samples described in this work, related to Figure 2 Only miRNAs for which the sum of cpm counts of all samples is greater than 100 are shown.

Document S2. Article plus supplemental information
