# Supplementary material for: In vivo AGO-APP for cell-type- and compartment-specific miRNA profiling in the mouse brain
Source: Cell Rep Methods. 2025 Dec 29;6(1):101267. doi: 10.1016/j.crmeth.2025.101267 (PMC12853173; doi:10.1016/j.crmeth.2025.101267)
Supplement: Document S1. Figure S1 [file mmc1.pdf]

Cell Reports Methods, Volume *6*

Supplemental information

In vivo AGO-APP for cell-type- and compartment-specific miRNA profiling in the mouse brain

Surbhi Kapoor, Andrea Erni, Francesca Vincenzi, Beatrice Tessier, Vasika Venugopal, Gunter Meister, Alexandre Favereaux, Harold Cremer, and Christophe Beclin

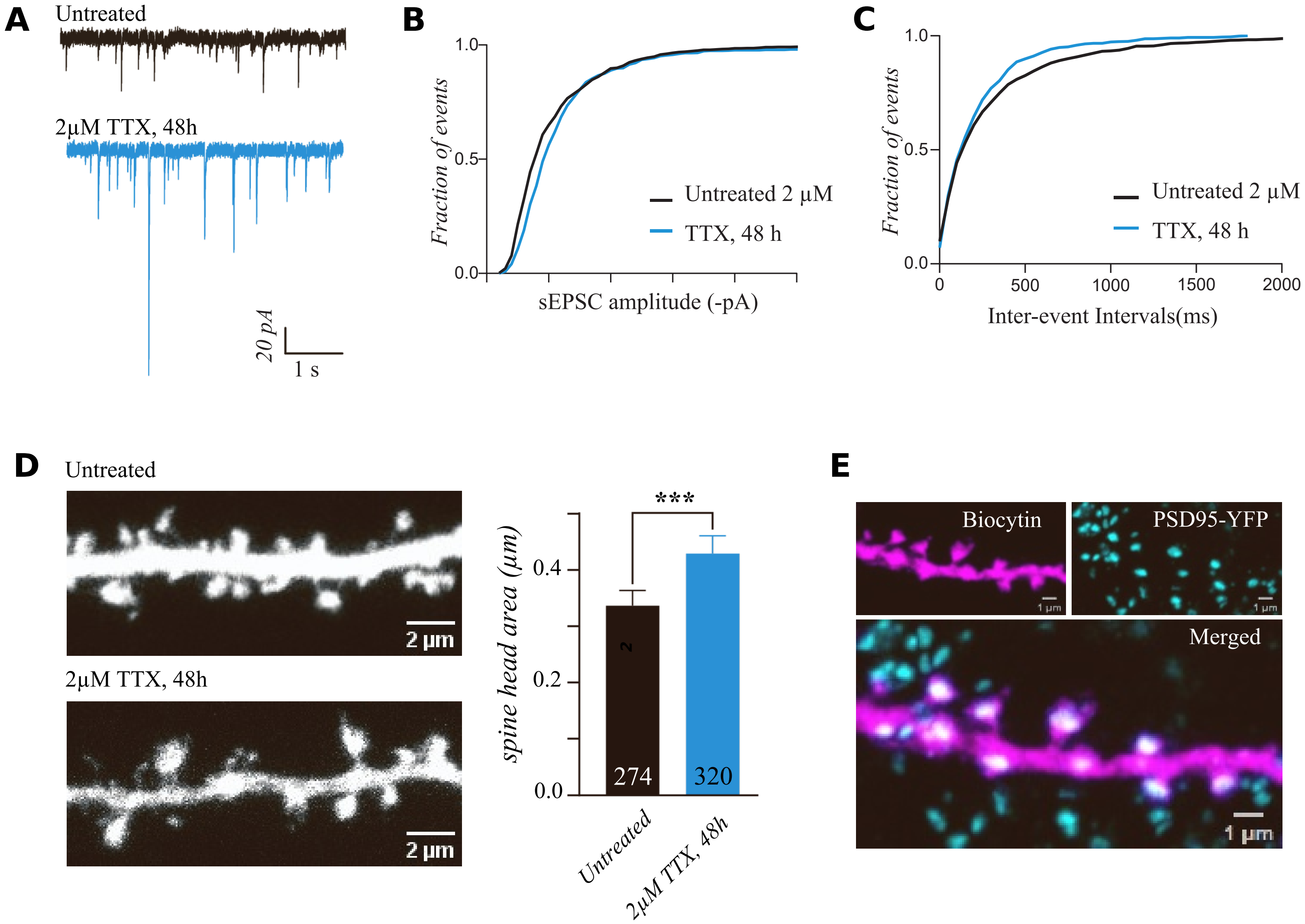

Figure S1. TTX induced homeostatic synaptic scaling in PSD95-T6B expressing hipocampal neurons. Related to Figure 3.
(A) Representative traces of AMPAR-mediated spontaneous synaptic currents (sEPSCs) recorded in pyramidal neurons from organotypic slices treated with 2 µM TTX for 48 h (blue) or left untreated (black). (B) Cumulative probability distributions of sEPSCs amplitudes for untreated neurons (black, n = 9 neurons from 4 slices) and TTX-treated neurons (blue, n = 5 neurons from 3 slices). Untreated vs TTX, p-value = 0.01 (Kolmogorov-Smirnov test). (C) Cumulative probability distributions of sEPSCs inter-event intervals for untreated neurons (black, n = 9 neurons) and TTX-treated neurons (blue, n = 5 neurons). Untreated vs TTX, p-value = 0.016 (Kolmogorov-Smirnov test). (D) Left: Confocal images of dendritic segments from untreated and TTX-treated pyramidal neurons filled with biocytin and labelled with AlexaFluor 647-conjugated streptavidin. Right: Average spine head for untreated neurons versus TTX-treated neurons. Numbers indicate the total number of dendritic spines analysed in each group (Untreated: 274 spines from 3 neurons from 2 slices; TTX treated: 320 spines from 5 neurons from 3 slices). (E) Confocal images of dendritic segments from TTX-treated pyramidal neuron filled with biocytin and imaged for PSD95-YFP.
Of note, these experiments were only performed in PSD95-T6B-FHY x NeuroD6-CRE-ERT2 animals as we did not access to exact control animals expressing PSD95-YFP.
